# Acute-Onset Iron Overload Cardiomyopathy in Diamond-Blackfan Anaemia: A Case Report of Rapid Ejection Fraction Decline

**DOI:** 10.7759/cureus.116110

**Published:** 2026-09-11

**Authors:** Joseph Waddon, Charlotte Hawkins, Mikaella Toquero, Sushmitha Thiagarajah Naidu, Munzir Malik

**Affiliations:** 1 Hospital Medicine, Wrexham Maelor Hospital, Wrexham, GBR; 2 Acute medicine, Wrexham Maelor Hospital, Wrexham, GBR

**Keywords:** cardiac mri iron loading, desferasirox, desferrioxamine, diamond-blackfan anaemia, ejection fraction decline, heart failure, iron chelation therapy, iron overload cardiomyopathy, transfusion-related anaemia

## Abstract

Diamond-Blackfan anaemia (DBA) is a rare inherited bone marrow failure syndrome causing pure red cell aplasia. Patients frequently require long-term red cell transfusions, predisposing them to iron overload. This case describes a patient with DBA with rapid ejection fraction decline secondary to acute-onset iron overload cardiomyopathy.

A 28-year-old male patient with known transfusion-dependent DBA and established iron overload and on deferasirox, presented with a one-week history of orthopnoea, dyspnoea and peripheral oedema. Echocardiography demonstrated severe biventricular impairment with an ejection fraction of 15-25%. This represented a marked decline from an estimated ejection fraction of 45% two years prior. His course was then complicated by fast atrial fibrillation and severe refractory hypokalaemia, leading to escalation to the critical care unit. Cardiac MRI confirmed significant myocardial and hepatic iron loading. He was then managed with intravenous deferrioxamine, heart failure pharmacotherapy and rate control, with subsequent escalation of oral chelation. He was then discharged with combined cardiology and haematology follow-up.

This case illustrates a severe complication of DBA: acute-onset iron overload cardiomyopathy causing rapid ejection fraction decline despite ongoing oral chelation. Markedly elevated ferritin, transferrin saturation and serum iron, alongside a normal CRP, supported a diagnosis of severe systemic iron overload. Management required a rapid switch from oral deferasirox to intravenous desferrioxamine.

This case demonstrates that severe, acute iron overload cardiomyopathy can occur in patients with transfusion-dependent DBA despite ongoing chelation. It supports the value of further study of cardiac outcomes in this patient group.

## Introduction

Diamond-Blackfan anaemia (DBA) is a rare inherited condition, that results in cellular defect in erythroid progenitors and precursors that are highly sensitive to death by apoptosis, this leads to erythropoietic failure [1]. In patients with clear evidence of red cell failure, characterised by macrocytic anaemia, reticulocytopenia and reduced or absent erythroid precursors in the bone marrow, differentials of pure red cell aplasia include DBA [1]. Clinical signs of DBA present in infancy, with approximately 90% of patients displaying symptoms in their first year of life [2]. The symptoms include predominantly anaemia-related symptoms such as pallor, fatigue, rapid heartbeat and weakness [1,2]. Congenital abnormalities, particularly cardiac and renal defects, as well as craniofacial malformations and short stature, can also be features of patients with DBA [3]. 

Patients with classic DBA often present within the first year of life with macrocytic anaemia without any other significant cytopenias, reticulocytopenia, and scarcity of bone marrow erythroid precursors and otherwise unaffected marrow architecture [4]. 

Patients with DBA are at high risk of developing malignancy and are predisposed to iron overload [3]. Iron overload cardiomyopathy is caused by damage to the musculature of the heart as a result of excess iron accumulation within the cardiac myocytes [5]. It occurs most commonly due to repeat red blood cell (RBC) transfusions but can also be due to hereditary haemochromatosis [5,6]. Excess intracellular iron induces oxidative stress and disrupts the normal calcium handling in cardiomyocytes, progressively impairing both the electrical conduction and contractile function of the heart [7]. 

Iron overload is a common complication of DBA; however, its relationship with iron overload cardiomyopathy is rarely reported in literature [8-10]. A PubMed search found minimal publications studying the relationship between DBA and iron overload cardiomyopathy [10]. Iron overload is a frequently expected complication in a patient who is transfusion dependent, but progression to clinically significant cardiomyopathy is rare [2,10]. It is predominantly reported by registry deaths or individual case reports. However, whether it is truly rare or poorly researched remains unclear. 

This case report addresses a gap in the literature by describing an instance of acute-onset iron overload cardiomyopathy in a patient with transfusion-dependent DBA established on oral chelation therapy, highlighting that clinically significant cardiac iron toxicity can arise acutely despite ongoing chelation.

## Case presentation

A 28-year-old male patient presented to the emergency department following a visit to his general practitioner (GP) with a one-week history of shortness of breath, and a four-day history of a non-productive cough. He denied chest pain, pleuritic pain or palpitations. He reported that the shortness of breath was worse when lying flat. He had noticed some bilateral leg swelling and was sleeping with four pillows at night. He had been started on 20 mg of oral furosemide once daily by the GP one week prior for suspected heart failure. He reported that since being initiated on furosemide, he had upped his oral fluid intake to around three to four litres per day, due to concerns about dehydration. He also reported some mild abdominal swelling, and the GP had noted that he was clinically mildly jaundiced. 

Medical history 

His past medical history included DBA, for which he received regular RBC transfusions. He received four units of RBCs every four weeks. He also had hypothyroidism, iron overload and type 2 diabetes mellitus. He was last transfused four days prior to admission with four units of RBC. 

The patient had taken deferasirox 2x360 mg tablets in the morning and 2x360 mg tablets in the evening. He had also taken 20 mg furosemide once in the morning and had no known drug allergies. 

Social history 

In terms of social history, he denied alcohol consumption and was an ex-smoker, which was not further quantified. He was independent and lived with his family. 

Observations 

Vital parameters at the time of presentation are given in Table 1.

**Table 1:** Observation at the time of presentation to emergency department SpO2: percentage of oxygen-carrying haemoglobin.

Parameter	Value	Reference range	Unit
Blood Pressure	116/82	Systolic 111-129 mmHg	mmHg
Heart Rate	116	51-90	beats/min
SpO2	96%	≥96%	% on air
Respiratory Rate	22	12-20	breaths/min
Temperature	37	36.1-38	degree celsius

Examination 

On auscultation, his heart sounds were dual with no added sounds; however his jugular venous pressure was elevated. Respiratory examination revealed reduced breath sounds to both bases, no expiratory wheeze and no crepitations. Abdominal examination revealed a soft abdomen with pitting oedema and present bowel sounds. There was also bilateral pedal oedema to the shins. Neurological exam was grossly normal, and the patient had a Glasgow Coma Scale score of 15/15. 

Laboratory investigations 

The complete set of investigations conducted upon the patient's arrival to the emergency department are shown in Tables 2, 3.

**Table 2:** Blood tests conducted upon arrival to the emergency department

Investigation	Value	Reference Range	Unit
Full Blood Count			
Haemoglobin (Hb)	76	120-160	g/L
Mean Corpuscular Volume (MCV)	90	80-100	fL
Platelets (Plt)	106	150-400	x10⁹/L
White Cell Count (WCC)	8.5	4.0-11.0	x10⁹/L
Neutrophils	4.7	1.8-7.5	x10⁹/L
Inflammatory markers			
C-reactive Protein (CRP)	3	<5	mg/L
Urea and Electrolytes			
Sodium	126	135-145	mmol/L
Potassium	4.8	3.5-5.0	mmol/L
Urea	12.4	2.5-7.8	mmol/L
Creatinine	89	60-110	µmol/L
Estimated Glomerular Filtration Rate (eGFR)	90	>60	mL/min/1.73m^2^
Liver Function Tests			
Bilirubin	25	<21	µmol/L
Albumin	41	35-50	g/L
Alkaline Phosphatase (ALP)	92	30-130	U/L
Alanine Aminotransferase (ALT)	141	<50	U/L
Bone profile			
Calcium (adj)	2.18	2.20-2.60	mmol/L
Phosphate	1.20	0.80-1.50	mmol/L
Albumin	41	35-50	g/L
Alkaline Phosphatase (ALP)	92	30-130	U/L
Iron studies			
Iron	63	10-30	umol/L
Ferritin	12612	15-300	ug/L
Transferrin	2.8	2.0-4.0	g/L
Transferrin saturation	89.6	20.0-50.0	%
Thyroid Function			
Thyroid Stimulating Hormone (TSH)	23.20	0.25-4.2	mU/L
Free T4	8.5	11-25	pmol/L
Other			
Lactate	1.8	0.5-1.6	mmol/L
N-terminal pro-B-type natriuretic peptide (NT-proBNP)	7202	<300	ng/L
High-sensitivity Troponin T	43	<14	ng/L
Magnesium	1.15	0.7-1.00	mmol/L
Amylase	29	<100	U/L
Osmolality	276	275-295	mmol/Kg

**Table 3:** Urinary investigations Reference ranges were not provided by Welsh Clinical Portal (WCP), the electronic clinical record system used within NHS Wales, and were therefore not applicable.

Investigation	Value	Unit
Osmolality	726	mmol/Kg
Sodium	<20	mmol/L
Potassium	36.4	mmol/L
Urea	500.6	mmol/L
Creatinine	9.90	mmol/L

Initial imaging

The chest X-ray at admission (Figure 1) revealed bilateral pleural effusions which were stable in appearance compared to previous imaging.

**Figure 1:**
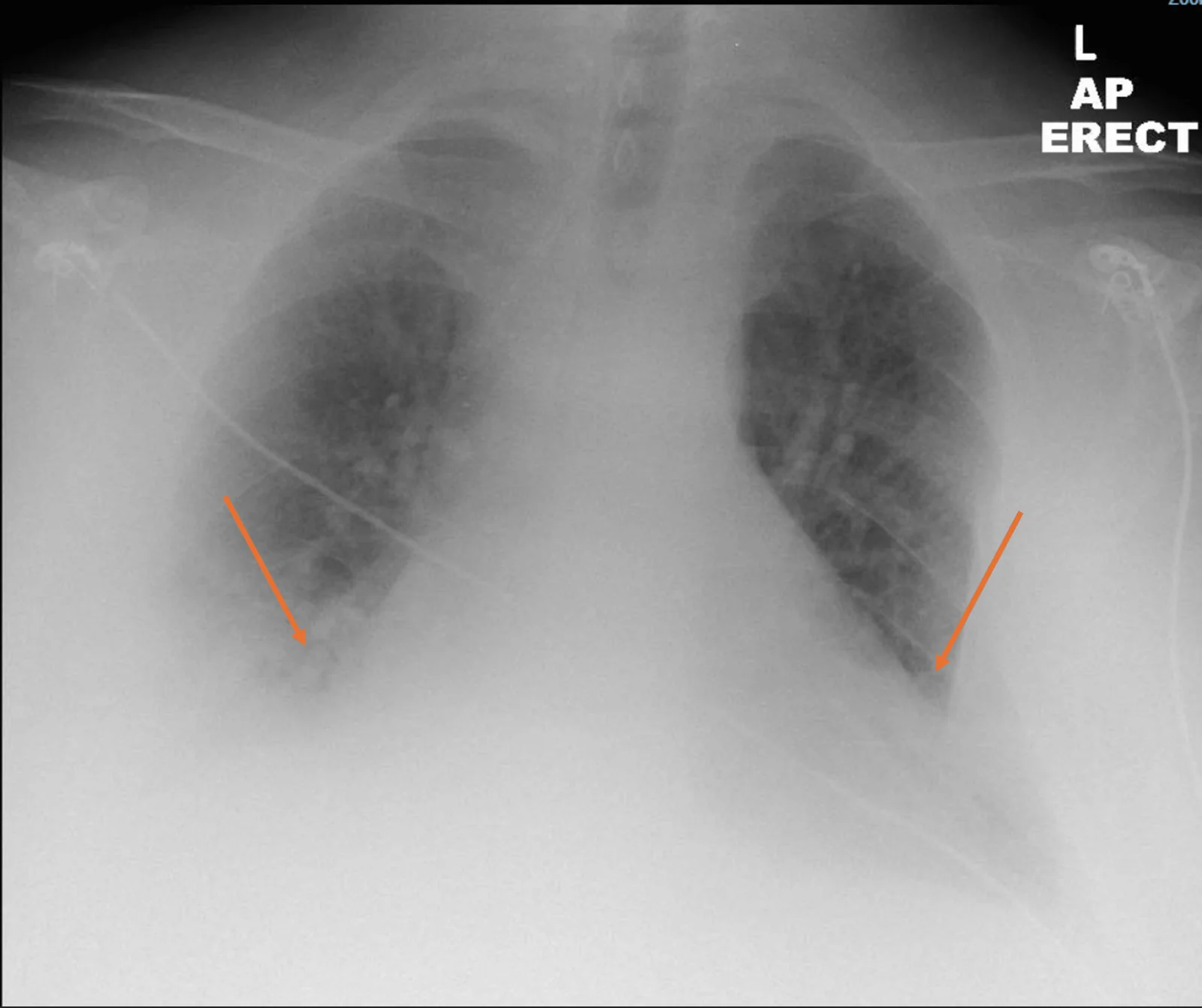
Chest X-ray at admission showing bilateral pleural effusions, indicated by the arrows

The remainder of the lungs were clear. However, it was an antero-posterior film and so cardiac size could not be commented on. 

Bedside echo was performed and revealed severe biventricular failure, with ejection fraction of ~15%. Moderate/severe mitral regurgitation (MR) was also found. The scope of this study was limited as the patient insisted on remaining in the chair, however the aforementioned findings were clearly visualised. 

Ultrasound abdomen revealed an enlarged and echogenic liver, consistent with fatty infiltration, with no focal lesions identified. The examination, within the scope of a practically difficult scan due to patient habitus and bowel gas, was otherwise grossly normal. 

Transthoracic echocardiogram

The patient’s first transthoracic echocardiogram was performed in mid-2024 (two years and four months prior to this admission). This revealed mildly impaired left ventricular systolic function with dysnergic anteroseptum normal dimensions estimated ejection fraction of ~45% and mild tricuspid regurgitation (TR) with peak gradient of ~31 mmHg possibility of raised pulmonary pressures. It also showed mild MR. No left ventricular hypertrophy (LVH) was noted. 

Video 1 shows a clip of the patient’s next transthoracic echocardiogram, which was performed on day 12 of admission, revealing severe left ventricular impairment globally with abnormal septal motion and D-shaped septum seen in both systole and diastole.

**Video 1:** Transthoracic echocardiogram from day 12 of admission, showing an apical view of severe left ventricular impairment

Estimated ejection fraction was approximately 20%. The right ventricle was dilated and impaired. The atria appeared dilated but no formal measurements. The mitral valve (MV) appeared normal with mild MR, no mitral stenosis (MS). The aortic valve (AV) was trileaflet and opened well, with no aortic regurgitation (AR) or aortic stenosis (AS). The tricuspid valve (TV) was abnormal in appearance with thickened leaflets and restricted leaflet opening. The flow through the TV was turbulent with severe TR noted. 

Management and hospital course 

The initial management included intravenous diuresis, referral to Haematology for guidance, fluid restriction of 1.5 litres and an urgent formal echo. The patients also underwent an ultrasound scan of his abdomen (results above) and was referred to endocrinology for input regarding his thyroid function tests. The haematology team felt that a transfusion of two units of RBCs was indicated, with IV furosemide between units due to the risk of transfusion- associated circulatory overload. 

Furosemide dose was escalated from 40 mg IV OD to an infusion of 240 mg in 24 hours, and the patient remained on a fluid restriction, strict input output monitoring, daily weights and daily blood tests to monitor renal function and electrolytes. 

The patient's care was transferred to the cardiology team, and he was commenced on heart failure with reduced ejection fraction management;. He was started on sacubitril with valsartan (Entresto), spironolactone and dapagliflozin. He was also started on IV desferrioxamine. 

His serum potassium level was persistently low, initially at 3.2 mmol/L and oral replacement was commenced but then worsened to 2.7 mmol/L, likely secondary to the furosemide infusion. Intravenous potassium replacement was administered in the smallest volume feasible, in order to minimise fluid load. However, despite this, the patient remained critically unwell and still remained at risk for a full escalation. 

A discussion about do not attempt resuscitation was conducted with the patient and his family, and it was agreed that he would not be resuscitated, but active treatment would continue. The potassium levels continued to drop, and repeats showed a serum level of 2.1 mmol/L. At this stage, the patient was escalated to the critical care team to takeover care.

The patient’s heart rate was also persistently high at around 150 beats per minute. He was initially given 5 mg IV metoprolol with limited effect and subsequently loaded on digoxin. The rate control was done cautiously in the context of the expectedly low blood pressure because of the furosemide infusion. ECGs showed fast atrial fibrillation with some T-wave inversion in leads V1 and V2 and he was started on a direct oral anticoagulant (DOAC) as per the cardiology team’s advice. 

The patient was transferred to the intensive care unit for central potassium replacement and blood pressure support, due to deteriorating hypotension. A cardiac MRI was arranged to look for iron loading in the myocardium, to happen once the patient was clinically more stable. The 24-hour urinary iron output was 1200 micromol/24hr. 

The cardiac MRI with iron loading revealed findings consistent with moderate cardiac iron loading and severe hepatic iron loading. The left ventricle showed global hypokinesia and severely reduced ejection fraction. The right ventricle showed diastolic flattening of the intraventricular septum in keeping with increased RV volume loading. 

The patient’s blood parameters were stable and was switched back to oral deferasirox, but the frequency was increased from twice daily to three times daily as per haematology advice. He was discharged with haematology and cardiology follow-up. 

## Discussion

The case report describes acute decompensated biventricular failure in a young man with transfusion-dependent DBA and severe systemic iron overload despite ongoing oral chelation therapy. The decline in left ventricular ejection fraction from approximately 45% in 2024 to 20-25% during this current admission demonstrates the progression of myocardial dysfunction. This case carries significant clinical importance as it illustrates a severe, but potentially modifiable, complication in a young adult with a rare inherited disease [2,11]. 

Chronic red-cell transfusion is the principal cause of iron overload in DBA [2]. Each unit of packed RBCs contain approximately 200-250 mg of iron and the body has no effective physiological mechanism of excreting this excess [2,12]. A patient receiving four units every four weeks may therefore accumulate approximately 12 g of transfusional iron each year if this schedule is sustained. Without adequate chelation, this burden progressively accumulates in the liver, heart and endocrine organs [2,12]. 

In a case-control study of 31 transfusion-dependent patient with DBA, Roggero et al. found severe iron overload 54% of them, particularly among patients with inadequate chelation [13]. Although this was a small cohort, it supports the understanding that patients with DBA may develop severe iron overload early and require careful monitoring of transfusion burden, iron status and response to chelation. 

In DBA, non-transferrin-bound iron is particularly important, because iron delivered through transfusion is not effectively utilised by the suppressed erythroid compartment. Porter et al. demonstrated higher non-transferrin-bound iron concentrations in transfused patients with DBA than in comparable patients with thalassaemia or sickle cell disease [14]. The 2024 international DBA consensus statement notes that non-transferrin-bound iron becomes increasingly important when transferrin saturation exceeds approximately 60-70% [2]. In this patient, transferrin saturation was 89.6%, with a serum iron of 63 µmol/L and ferritin of 12,612 µg/L. These results provide strong evidence of severe systemic iron overload and suggest that potentially toxic circulating iron was present. The normal CRP makes inflammation alone an unlikely explanation for the markedly elevated ferritin. Nevertheless, ferritin and transferrin saturation cannot determine the amount of iron deposited within the myocardium and should not be used as substitutes for direct cardiac iron assessment. 

The use of point-of-care ultrasound (POCUS) was particularly valuable in this case. It demonstrated severe biventricular failure with an estimated ejection fraction of approximately 15%, providing an immediate cardiac explanation for the patient’s marked congestion. The findings supported escalation of diuretic therapy, cardiology involvement and definitive cardiac imaging. The focused cardiac ultrasound intends to provide an answer specific to clinical question at the bedside and can rapidly identify major abnormalities. However, it remains an adjunct to clinical assessment and does not replace comprehensive echocardiography [15]. 

Although comprehensive echocardiography provided a detailed assessment of the ventricular and valvular abnormalities, it could not establish myocardial iron deposition or confirm iron-overload cardiomyopathy. Cardiac MRI was performed and particularly important because serum ferritin correlates poorly with cardiac iron burden. The international DBA consensus recommends MRI-based cardiac iron assessment rather than reliance on ferritin alone when evaluating iron burden and guiding chelation therapy [2]. 

The management of this patient’s iron overload was escalated rapidly during admission; switching from oral deferasirox to intravenous desferrioxamine, and finally with a frequency increase of the oral iron chelation therapy on discharge. Intravenous desferrioxamine is often seen to be favoured in acute, severe iron overload as it can be administered continuously [2]. However, there is very limited evidence on chelation strategies in the context of acute iron-overload cardiomyopathy in DBA. Current chelation strategies are extrapolated from the experience of other transfusion-dependent conditions, such as thalassaemia [12,16,17]. DBA is comparably a rarer disease and thus no equivalent body of evidence exists and may have a differing iron-handling profile [2,14]. 

Iron-overload cardiomyopathy should be considered early when a transfusion dependent patient with DBA develops unexplained breathlessness, arrythmias even with ongoing iron chelation therapy. The international DBA consensus recommends annual MRI-based liver and cardiac iron assessment in a chronically transfused patient [2]. 

It must be acknowledged that the acute nature of this cardiomyopathy was likely multifactorial. While iron overload, supported by the markedly elevated ferritin, transferrin saturation and MRI findings, is considered the predominant driver, other factors including hypothyroidism, atrial fibrillation and repeated transfusions may have contributed to the acute decompensation.

This case adds to the limited literature describing chelation strategies and escalation of chelation in the context of acute DBA cardiomyopathy. It highlights the current literature gap of the absence of dedicated papers for DBA-specific chelation intensity, route, or duration in the context of acute cardiac decompensation and clinicians are reliant upon extrapolated protocols. 

This case report is notable for multiple reasons. Iron overload is a well-acknowledged complication of patients with DBA who are transfusion dependent, but progression to clinically significant iron-overload cardiomyopathy is rare. A targeted, extensive literature search revealed limited case-based literature focusing on this association. The lack of published findings likely reflects a combination of under-recognition or under-reporting and true rarity. The existing literature is primarily compiled of case reports and mortality data, rather than systematic studies. Another notable aspect of this case is the severity and acuity of the presentation; the development of acute biventricular failure with ejection fraction of 15-20% and subsequent haemodynamic instability requiring critical care support, over a relatively short timeframe. This contrasts with the well-recognised description of a slow, progressive decline seen in iron-overload cardiomyopathy [16]. 

## Conclusions

This case depicts how severe, acute iron overload cardiomyopathy can be seen in patients with transfusion-dependent DBA despite ongoing chelation therapy. Moreover, its presentation may be more acute in onset and severe in nature than typically described. There is an apparent gap in established literature to guide chelation intensity and duration in DBA-specific acute iron-related cardiac decompensation, with current practice largely extrapolated from other transfusion-dependent conditions. This case report supports the need for systematic studies of cardiac outcomes in patients with transfusion-dependent DBA to help identify the true incidence of iron overload cardiomyopathy in this patient group and inform specific evidence-based practice. 
